# Supplementary material for: The prevalence of age-related macular degeneration and osteoporosis in the older Polish population: Is there a link?
Source: PLoS One. 2023 Oct 19;18(10):e0293143. doi: 10.1371/journal.pone.0293143 (PMC10586687; doi:10.1371/journal.pone.0293143)
Supplement: Attachment — Submitted filename: Review_1st.docx [file pone.0293143.s001.docx]

**Comments to the Authors.**

This handles an interesting theme regarding the association between the osteoporosis and AMD. There are several comments and questions.

**Major points**:

1. How relevant the findings are for practitioners and patients? That is, what impact will the findings have on the current clinical practice? The authors should discuss about it.
2. Line 337: “As the risks of AMD and osteoporosis both increase with age, this factor may explain the lack of a significant relationship between AMD and low BMD in women in this study.”
   This explains why there was no significant association between AMD and osteoporosis for women. What do the authors think about only men having significant association? The findings seem to be opposite to the previous reports from the USA and Korea the authors cited.
3. Were there any data on cataract, glaucoma and diabetic retinopathy? The association between these disorders and osteoporosis would be valuable, which would clarify if the current findings are a disease-specific association, that is, if only AMD is associated with osteoporosis.

**Minor points**:

1. Introduction is too long. The authors might want to reduce it below 300 words. General explanation of the disease would be unnecessary like “Clinically, osteoporosis manifests as a higher risk of skeletal fragility and apparent major osteoporotic fractures that frequently lead to disability, mobility restriction, decreased quality of life, and premature mortality.”
2. Line 223-233: “the predictors were age and crude femoral BMD in one (Table 5), and age and femur T-score in the other (Table 6).” This should be removed to the Methods section.
3. The authors should describe why the threshold (T-score = -0.1) was selected (e.g. it’s clinically important, often used, etc.).
4. What is the difference between the “Total femur T-score” in the bottom row of Table 1 and “T-score≥-0.1” or “T-score<-0.1” in Table 2? Is something just categorized “Total femur T-score” into used in Table 2? Why did the authors take the trouble to do so? This is related to the comment 3.
5. I couldn’t understand the part of Nagelkerke pseudo-R^2^. The authors should explain how to interpret this statistic value for readers to be able to understand easily in the Methods section (not in the Results section).
6. I think the following paragraph is redundant and not necessary (lines 265-272): “The first manifestation of AMD is damage to the retinal pigment epithelium (RPE), a monolayer of cuboidal, pigmented epithelial cells located between the neurosensory retina and the choroid. It has a plethora of functions, including delivering blood-derived nutrients to photoreceptors; transporting ions, water, and metabolites from the subretinal space to the blood; absorbing light; phagocytizing photoreceptor outer segments; supporting recycling of all‐ trans retinal back to 11‐ cis retinal; secreting neurotrophic factors; and scavenging damaged reactive oxygen species [19]. Thus, functional abnormalities in the RPE lead to impaired function of the entire neural retina.”
7. I think the following paragraph is so long and should be made short (lines 273-294): “The pathogenesis of AMD is multifactorial, and the molecular mechanisms underlying its development are still not understood. Its pathogenesis involves environmental and genetic factors, disorders of the complement system, lipid abnormalities, angiogenic and inflammatory pathways, and the extracellular matrix [19,20]. A growing body of evidence suggests that oxidative stress and impaired protein degradation and clearance pathways in RPE cells may lead to their functional impairment and damage. Autophagy plays a key role in eliminating cellular waste materials, such as aggregated proteins and dysfunctional cell organelles, from RPE cells. RPE cells cultured from donors with AMD are reportedly more sensitive to oxidative stress and impairment of autophagy than RPE cells from donors without AMD [21]. Autophagy is vital for the survival of cells under stress, especially RPE cells, given their metabolic activity and paucity of physiological functions. Impairment of autophagy leads to lipofuscin accumulation in RPE cells and deposition of lipoproteins, in the form of drusen, in the extracellular spaces. Drusen formation is the first manifestation of one of the two forms of AMD, namely, dry AMD (dAMD). dAMD is also characterized by RPE dysfunction and photoreceptor loss. In approximately 10%–15% of cases, dAMD progresses to wet AMD (wAMD), characterized by choroidal neovascularization with intra- and subretinal shunting, hemorrhages, and RPE detachment. wAMD can be treated with intravitreal injections of antiVEGF agents. Although this form of AMD accounts for only 10%–15% of all AMD cases, it is the leading cause of substantial eyesight deterioration and the principal source of expenditures associated with treatment for AMD [21,22]. A conservative estimate of the cost of wAMD treatment in the USA was demonstrated to generate a cost of $1.1 billion for the entire population in year 1 and $5.1 billion in year 3 [22].”

   The next paragraph refers to “autophagy”, which is only the element needed in this paragraph (lines 273-294).
8. Line 342: “Nonetheless, even considering that the vertebral BMD in these individuals remained unaffected, a lower femoral BMD may increase the risk of osteoporosis and subsequent hip fractures in men”
   I couldn’t understand this sentence. I thought the authors investigated the association between AMD and osteoporosis (T-score), not hip fractures. Did the authors look into hip fractures?
